# Deconstructing the OSCE

**DOI:** 10.1192/pb.bp.115.052829

**Published:** 2017-02

**Authors:** Deborah Cooper

This volume contests that it is possible to develop a generic way to approach different Observed Structured Clinical Examination (OSCE) scenarios, regardless of medical specialty. Perhaps surprisingly, it is not filled with possible exam scenarios, but rather looks at the underlying barriers to good performance. In this regard, it provides a behavioural and psychological schema for approaching the OSCE. The book makes no apologies for aiming at those who have already had a previous attempt at passing the OSCE, and given that pass rates for membership exams are generally around 50–60%, it is a resource available to a great number of doctors in training.

The initial chapters look at the common emotional and cognitive responses which typically follow an unsuccessful examination attempt – they do a good job of validating these experiences and feelings. Subsequent chapters aim to improve general exam strategy. These include the perhaps more neglected areas of good exam performance; for example, how to establish rapport with the actor or patient, and how to run a good study group and learning environment. An especially useful chapter is that which explores challenging scenarios such as ‘the angry relative’ or ‘the crying patient’. Although these passages are brief, practical tips are given to aid communication in these often difficult situations.

In addition, there are worksheets that support the doctor in understanding that the way they think about the exam influences their emotions and, ultimately, their exam performance. The psychiatry trainee will be no stranger to this process; however, I wonder whether trainees from other specialties might find the experience alienating.

This work undoubtedly highlights that poor exam performance is often not related to lack of knowledge, but to cognitive and emotional barriers. As a result, it may provide a good starting point for ongoing study where examination performance has proven problematic.

